# Changes in metazoan functional diversity across the Cambrian Radiation and the first Phanerozoic mass extinction: the Cambrian Sinsk Event

**DOI:** 10.1098/rspb.2025.0968

**Published:** 2025-08-20

**Authors:** Adam Murphy, Amelia Penny, Andrey Zhuravlev, Rachel Wood

**Affiliations:** ^1^ School of Geosciences, University of Edinburgh, Edinburgh, Scotland, UK; ^2^ Borissiak Palaeontological Institute, Russian Academy of Sciences, Moscow, Russia

**Keywords:** Cambrian Radiation, functional diversity, mass extinction, Sinsk Event

## Abstract

The Sinsk Event (approx. 513.5 million years ago, Ma) is the first Phanerozoic mass extinction, marking the end of the canonical Cambrian Radiation. We reconstruct taxonomic and functional diversity patterns of skeletal metazoans from the Siberian Platform during the Cambrian Radiation and across the Sinsk Event from approximately 529 Ma to 508 Ma, to investigate the changing occupation of functional space and the evolution of functional traits during the radiation, and the role of these in extinction selectivity at the Sinsk extinction and subsequent recovery. During the radiation, functional richness increased before taxonomic richness as new groups with novel traits emerged and diversified. Taxonomic richness declined sharply at the Sinsk, but thereafter increased rapidly while functional richness continued to decline until approx. 508 Ma, indicating a post-extinction decoupling. While there is limited evidence of extinction selectivity at the Sinsk, certain functional traits are associated with post-extinction recovery from approximately 511 Ma to 508 Ma. Groups with novel functional traits associated with motility, diversified feeding modes and broad water depth tolerances diversified rapidly, while sessile, inshore filtrators and heavily calcified taxa which had been dominant prior to the extinction either failed to recover or became extinct. The Sinsk Event therefore marks a significant transition in marine ecosystem function.

## 1. Introduction

The Cambrian Radiation marks the appearance of diverse and abundant skeletal animals (metazoans), mostly bilaterians, in the fossil record. Many new taxa and functional groups originated during this interval, which was also marked by extremely high rates of extinction and turnover [1–3]. The diversification during the Cambrian Radiation may have been driven by oscillating ocean oxygenation events (OOEs) [4–7], but was terminated by the first global mass extinction of the Phanerozoic.

**Figure 1.**
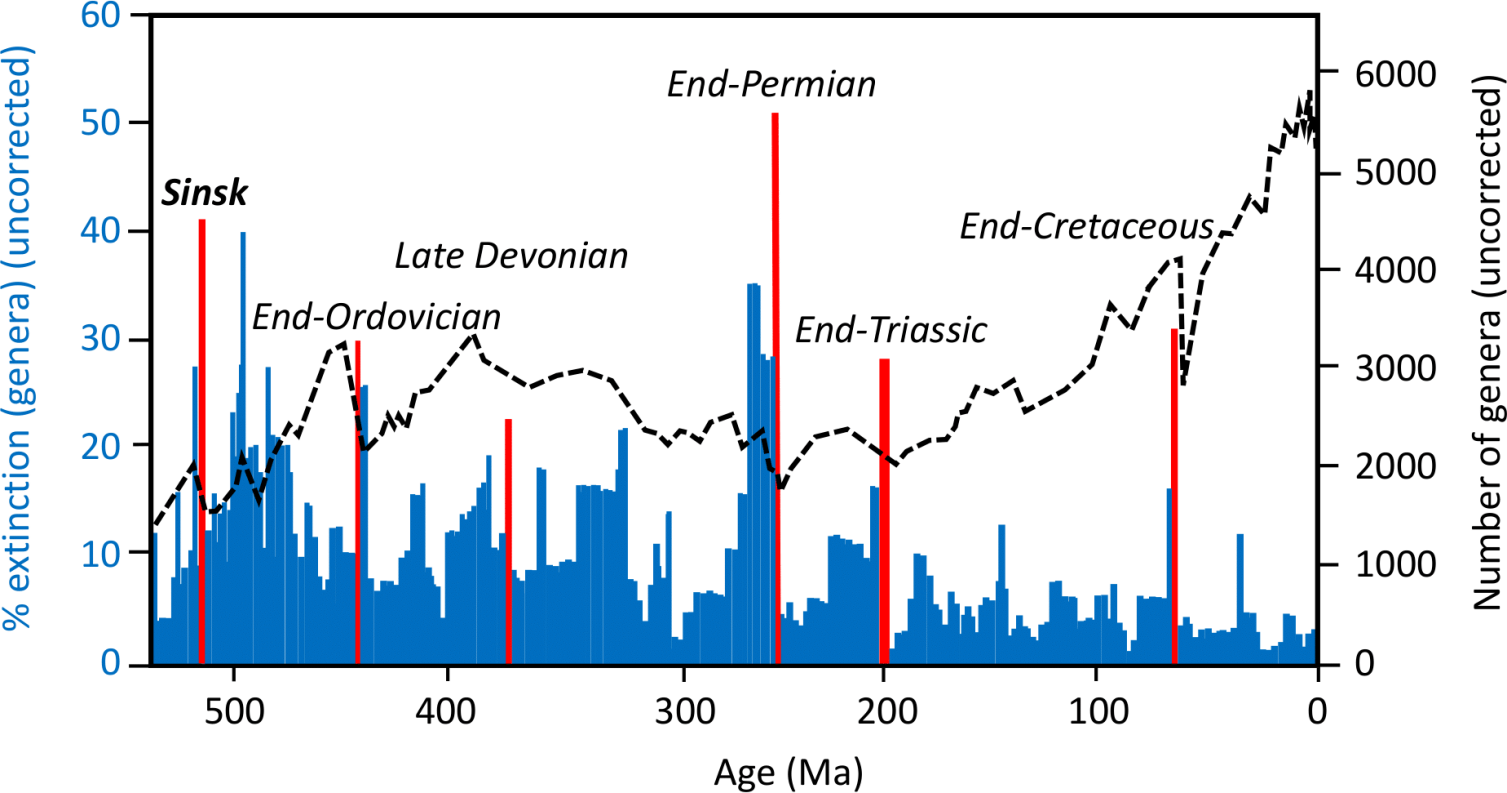
Phanerozoic marine metazoan diversity (dotted line, right *x*-axis; number of genera per international geological stage bin, uncorrected for sampling) and generic extinction intensity (all histograms, left *x*-axis; fraction of genera present in an interval but not in the subsequent interval, uncorrected for sampling), showing the big five mass extinctions and the Sinsk Event (red histograms). Modified from Raup & Sepkoski [2], Rohde & Muller [3] and Sepkoski [8].

Early studies (e.g. [2]) noted that high extinction rates (although uncorrected for sampling) were present in the early Botoman (early Stage 4) of the early Cambrian, but also that the absolute number of genera during the Cambrian was low compared with the rest of the Phanerozoic. As a result, this extinction was never included among the major mass extinctions of the Phanerozoic, which currently number five (figure 1). This peak of high extinction rates in the early Stage 4 of the Cambrian is now known as the ‘Sinsk Event’, occurring at approximately 513.5 Ma and named after the Sinsk Formation on the Siberian Platform where it is most clearly expressed [9]. Despite high extinction rates at the Sinsk Event (in terms of taxonomic change) comparable with subsequent mass extinctions events (e.g. end-Ordovician, Late Devonian), this event has received limited scrutiny or recognition.

The Sinsk Event was global, with dramatic reductions in metazoan diversity documented on the Siberian Platform, Mongolia, Laurentia, Morocco, Spain, Sardinia, Australia and South China [9,10]. The Sinsk Event removed or notably reduced the diversity of many typical Cambrian groups, such as reef-associated archaeocyath sponges and diverse small shelly fossils. Over 60% of metazoan species were lost globally, and metazoan biodiversity did not recover to pre-Sinsk Event levels until the early Ordovician [11–14]. As a result, the survivors of the Sinsk Event diversified into communities with dramatically different taxonomic compositions to those which had dominated before [15]. The Sinsk Event therefore marks the end of the canonical Cambrian Radiation, resetting the trajectory for subsequent metazoan evolution.

The Sinsk Event is marked by a global increase in the abundance of unbioturbated black shale [9], and a shift from coupled to uncoupled carbon and sulfur isotopic records has been noted on the Siberian Platform [4]. These all suggest that a decline in global oceanic oxygen levels creating an anoxic event played a major role in the extinction [4], but potential environmental triggers remain poorly understood. The Sinsk Event coincided approximately with emplacement of the Kalkarindji Large Igneous Province, which probably occurred at approximately 514–513 Ma [10,16,17]. This may have induced rapid global ocean warming creating a hyperthermal interval promoting widespread ocean anoxia [18,19]. The Sinsk Formation and similar coeval strata, which cover in total over 750 000 km^2^ on the Siberian Platform and contain extensive black organic- and disseminated pyrite- and uranium-rich shales, are coincident with the loss of many metazoan species and even trace fossils, as well as with abundant biomarker evidence for the presence of anaerobic bacterial communities [9,20–23]. The behaviour of carbon and carbonate-associated sulfur isotopes is inferred to indicate a reduction of oceanic sulfate concentrations and shoaling of anoxic waters onto shallow shelf settings during the Sinsk Event [4]. Indeed, the Sinsk Event occurred during a major marine transgression [6,24], and an upwelling of deeper anoxic waters onto shallower shelf areas is likely to have been the primary driver of the extinction [4,5,9,15,25]. Although organic-rich shales persisted until approximately 507 Ma (upper Amgan on the Siberian Platform = middle Wuliuan of the ICC, 2023 [26]), the duration of the anoxic interval itself is unknown.

Data of trilobite distribution from the Ross Orogen, Antarctica, and the Delamerian Orogen, Australia, across the Sinsk Event interval record synchronous and abrupt pulses of contractional supracrustal deformation on both continents [10]. As a result, it has been suggested that the Sinsk extinction was triggered by supracrustal contraction along the edge of Gondwana, which created a series of dramatic changes that included the loss of shallow marine carbonate habitats, extrusion of the Kalkarindji large igneous province with the release of large volumes of volcanic gases, so causing rapid global warming in turn creating the development of widespread shallow marine anoxic oceans.

Despite the fact that the Sinsk Event fundamentally altered metazoan communities in terms of taxonomic composition, little is known as to how functional diversity influenced extinction selectivity or the post-extinction restructuring of those communities. The functional traits of individual taxa and the communities to which they belong are instrumental in determining both how they are affected by perturbations and also how they respond in the aftermath [27]. Here, we reconstruct a high-resolution record of functional diversity in early Cambrian skeletal metazoans of the Siberian Platform through the Cambrian Radiation and the Sinsk Event to determine the likely controls on susceptibility to extinction. During the early Cambrian, the Siberian Platform was a vast, isolated tropical continent dominated by shallow marine carbonate settings that supported a single metacommunity, forming a biodiverse hotspot that supported a third of all documented early Cambrian skeletal metazoans [4,28].

Functional diversity (or ‘functional variety’ [29]) concerns the broad range of organismal traits which are assumed to influence communities and the ecosystems of which they are a part [29–31]. Quantifying changes in functional diversity within communities enables exploration of the impacts of environmental drivers on ecosystem function, which is not possible from taxonomic diversity patterns alone. Comparing taxonomic and functional diversity patterns also allows investigation of the links between functional space occupation and taxonomic diversity (e.g. [32–34]). While the occupation of functional space is broadly correlated with increases in marine taxonomic diversity across the Phanerozoic [34,35], the nature of this relationship at smaller temporal and spatial scales may be highly variable and context-dependent, and can be disrupted and decoupled during times of extreme perturbations such as mass extinction events (e.g. [36,37]). Functional diversity (and other measures of ecological functioning such as trophic webs) can take far longer than biodiversity to recover to pre-extinction levels of complexity [38–42]. Mass extinctions have also been shown to exhibit ecological selectivity (e.g. [36,43]), where taxa possessing particular traits are more likely to become extinct during a perturbation. Susceptibility and resistance to extinction pressures can thus be evaluated in studies which incorporate species traits.

Here we investigate the relationship between taxonomic diversity and the occupation of functional space. We characterize key traits with respect to ecological attributes, interactions and ecosystem functioning. This allows exploration of the decoupling of taxonomic and functional diversity, and the role of traits in extinction selectivity across the Sinsk Event. We hypothesize that the increase in taxonomic diversity prior to the Sinsk Event was correlated with the expanding occupation of functional space, but that these became decoupled at the Sinsk Event. We also suggest that recovery after extinction at the Sinsk Event was selective, and associated with particular traits.

## 2. Methods

We used a dataset of metazoan species on the Siberian Platform during the early Cambrian from approximately 529 Ma to 508 Ma (covering the Tommotian to Amgan stages following the Siberian timescale) from the Pestrotsvet, Perekhod and Sinsk formations [15,25]. Species occurrences in twenty taxonomic groups (table 1; electronic supplementary material, table S2) were collated and allocated to a series of 12 time bins following biostratigraphic zones with an inferred duration each of 0.7–4 Myr following the age model of Bowyer *et al*. [6]. Assemblages are defined here as all of the taxonomic groups present within a given time bin.

**Table 1.** Lower Cambrian Siberian Platform skeletal metazoan groups included in this study, with inferred phylogenetic affinity and summary of ecology and morphology. Note that while the vermiform groups Palaeoscolecida and Xenusia are described separately in this table, they were combined in a single taxonomic group for analyses.

taxonomic group	affinity	inferred ecology and morphology
Archaeocyatha	Porifera	reef-associated, attached, sessile; filter-feeders; conical or cup-like porous skeleton composed of high-Mg calcite [44–46]
Radiocyatha	possible Porifera	reef-associated, attached, sessile; filter-feeders; widely conical or subspherical porous skeleton composed of aragonite [44,45]
Cribricyatha	unknown	reef-associated, attached, sessile habit; filter-feeders; cup-like skeleton composed of high-Mg calcite [44,45]
Coralomorpha	possible Cnidaria	reef-associated, attached, sessile; filter-feeders; cup-like skeleton composed of high-Mg calcite [11,44]
Anabaritida	possible Cnidaria	unattached benthos; suspension-feeders; conical, tube with trifold symmetry and skeleton composed of aragonite [47,48].
Hyolithelminthida	possible stem-group Lophotrochozoa	attached benthos; suspension-feeders; conical, tube with skeleton composed calcium phosphate [49,50]
Palaeoscolecida	Ecdysozoa, stem-group Cycloneuralia [51]	semi-infaunal motile benthos; predators; vermiform skeleton composed of phosphatic sclerites [52,53]
Xenusia	Lobopodians, stem-group Euarthropoda [54]	epifaunal motile benthos; predators [55]
Trilobita	Ecdysozoa, Arthropoda, stem-group Mandibulata [56,57].	epifaunal motile benthos; predators and/or scavengers; plated exoskeleton composed of low-Mg calcite [44,53]
Bradoriida	Ecdysozoa, stem-group Euarthropoda [58]	epifaunal motile benthos; filter-feeders; skeleton composed of phosphatic sclerites [44,53]
Halkieriida	Lophotrochozoa, stem-group Mollusca [59]	epifaunal motile benthos; skeleton composed of aragonite [45,52]
Helcionelloida	Lophotrochozoa, stem-group Mollusca [60,61]	epifaunal motile benthos; suspension-feeders and grazers; single conchiferan shell composed of aragonite [62,63]
Bivalvia	Lophotrochozoa, Mollusca	infaunal motile benthos; suspension-feeders; bivalved shells composed of aragonite sclerites [44,45]
Tommotiida	Lophotrochozoa, stem-group Lophophorata [64]	epifaunal sessile and motile benthos; filter- and deposit-feeders; sclerites composed of calcium phosphate [45,65]
Hyolithomorpha	Stem-group Lophotrochozoa [66]	epifaunal motile benthos; suspension-feeders and scavengers; bivalved shell possibly composed of low-Mg calcite [44,45,52]
Orthothecimorpha	Stem-group Lophotrochozoa [67]	epifaunal and semi-infaunal sessile benthos; suspension-feeders; conical shells composed of aragonite [44,45,52]
Stenothecoida	Stem-group Lophotrochozoa [68]	epifaunal sessile benthos; suspension-feeders; bivalved shells composed of calcite [44,52]
Protoconodonta	Protostomia, probable Chaetognatha [69]	predatory nekton; vermiform with complex phosphatic tooth elements [45,70]
Silicea	Porifera (Demospongea, Hexactinellida and Silicea stem-groups)	sessile benthos; filter-feeders; spicules composed of silica [44,52]
Linguliformea	Lophotrochozoa, Brachiopoda	sessile benthos; suspension-feeders; bivalved shells composed of calcium phosphate [45,52]
Rhynchonelliformea	Lophotrochozoa, Brachiopoda	sessile benthos; suspension-feeders; bivalved shells composed of low-Mg calcite [45,52]

Functional diversity approaches generally involve the evaluation of trait-based differences among species or other taxonomic groups [71,72]. These traits may include any behavioural, morphological or physiological properties of an organism which are amenable to direct measurement (e.g. body size) or categorization within a set of defined ‘characters’ or ‘modalities’ (e.g. filter, suspension and predatory modes of feeding) [35,73]. The combined variation in trait modalities among the taxa studied can then be used to place them relative to one another on the basis of trait differences within a ‘functional space’ [31].

We selected functional traits which we expected to be potentially ecologically important for Siberian Platform metazoan communities, measurable from fossil specimens, and with modalities which could be assigned from the published literature (table 1). Each taxonomic group was assigned a set of trait modalities. Eight traits were analysed (see electronic supplementary material, table S1), of which six are associated with habitat and mobility (substrate relationship; motility; attachment) and ecological characteristics (feeding mode; full depth range; median depth; the depth range and median depth categories are based on [52]; electronic supplementary material, figure S4). Two skeletal traits were included: mineralogy (aragonite; high-Mg calcite; low-Mg calcite; silica; phosphate) and type (massive; cone; sclerite; single shell; bivalved shell; spicule; tooth). Skeletal traits may be associated with the capacity to physiologically buffer in low oxygen conditions, following the proposal of Knoll *et al*. [74]. An additional trait, body size, was not included since body size changes in skeletal metazoans on the Siberian Platform during this interval has previously been investigated by Zhuravlev & Wood [15].

Functional diversity analyses were performed in the R environment (version 2.2.2) using the package mFD (multifaceted functional diversity [75,76]; see electronic supplementary material). Trait-based distances between taxonomic groups were calculated using the Gower distance metric, since this can accommodate both continuous and categorical variables [77].

Principal coordinates analysis (PCoA) was used to calculate Euclidean distances between taxonomic groups based on the pairwise Gower distances calculated from the functional traits of all taxonomic groups across the study interval. A convex hull was then constructed for each assemblage, and functional richness and functional dispersion was calculated. Functional richness quantifies the proportional occupation of all possible functional space, while functional dispersion is the centroid between taxonomic groups in functional space, calculated from a species richness weighting. Functional beta-diversity between assemblages was also calculated to evaluate the degree of functional nestedness and turnover between assemblages. These functional diversity metrics were then compared with taxonomic diversity, which is a count of the number of taxonomic groups in each time bin.

## 3. Results

### (a) General metazoan group dynamics

**Figure 2.**
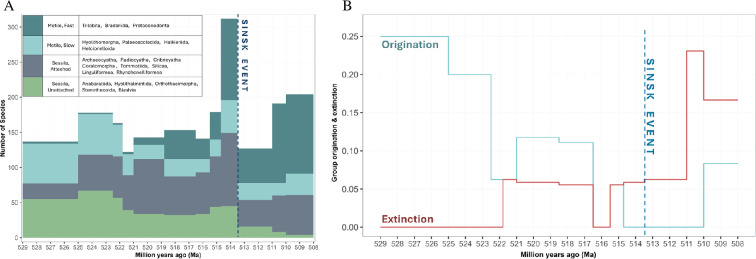
Skeletal metazoan dynamics on the lower Cambrian Siberian Platform from 529 Ma to 508 Ma, binned by biostratigraphic zones, with timing of the Sinsk Event. (A) Species richness, grouped by motility and attachment traits. (B) Rates of origination and extinction of the 20 studied groups. Extinction and origination rates are calculated as proportions of the total number of taxonomic groups present in each biostratigraphic zone.

Ten of the taxonomic groups considered through the approximately 529 Ma to 508 Ma interval (table 1; figure 2A; electronic supplementary material, figures S1 and S2) are relatively species-poor, with <10 species in any individual time bin. These include the reef-associated cribricyaths, radiocyaths and coralomorphs, as well as small shelly fossil groups (anabaritids, palaeoscolecidans, bradoriids, halkieriids, bivalves, stenothecoids and protoconodonts). Other groups are more diverse, notably archaeocyaths and trilobites, although is possible that here some taxonomic over-splitting is present [15]. Most groups decline in species richness by 521 Ma, and while some diversify again until the Sinsk Event (helcionelloids and hyoliths) others never recover their former diversity (anabaritids, tommotiids and hyolithelminthes). Linguliformean and rhynchonelliformean brachiopods appear by 521 Ma but retain low species richness until the Sinsk, after which they diversify with both groups reaching their peak diversities from 510 Ma to 508 Ma. Silicean sponges (Hexactinellida and Demospongea, together with their stem-groups) experience rapid diversification immediately post-Sinsk before declining thereafter. Trilobites undergo two phases of rapid diversification: one from approximately 515 Ma to the Sinsk at approximately 513.5 Ma, and a second phase immediately after the Sinsk. This pattern is reflected in both global cumulative diversity and the disparity of early Cambrian trilobites [78]. Helcionelloid molluscs and hyoliths also begin to diversify again by approximately 510 Ma, albeit far more slowly than trilobites. Only one taxonomic group (the Tommotiida) is lost in the immediate aftermath of the Sinsk, though a further four groups (Archaeocyatha, Coralomorpha, Halkieriida and Hyolithelminthida) while decimated by the Sinsk Event, did not disappear until approximately 510 Ma. Trilobites, helcionelloid molluscs, protoconodonts, hyolithomorph hyoliths and linguliformean and rhynchonelliformean brachiopods all diversify post-Sinsk Event.

The origination of new groups is episodic, occurring during the intervals approximately 529–523 Ma, 521−517 Ma and 515.5−514.5 Ma, but with origination rates declining successively, while extinction rates conversely show intervals of stepped increases, from 522−516.5 Ma to 515−511 Ma to 511−510 Ma (figure 2B). In sum, while overall group diversity was similar at the beginning and end of the early Cambrian studied interval, group composition was dramatically different.

### (b) Delineation of functional space

Pairwise Gower distances reveal that most taxonomic groups are quite distinct from one another in terms of trait values, with a mean Gower score among all groups of 0.7 (where a distance score of 1 indicates that two groups have different modalities for every trait, and 0 indicates identical modalities for every trait).

The initial PCoA generated 10 functional axes (which is the maximum calculable number of axes). Mean absolute deviation (MAD) scores indicated that the functional space with minimal deviation from the Gower distances was constructed with seven functional axes (a seven-dimensional space: MAD = 0.05 – well within the quality threshold of MAD = 0.1; electronic supplementary material, table S3). Each of the seven functional axes explains additional variance in the data (indicated by eigenvalues; electronic supplementary material, table S3), though the MAD scores begin to rise with the inclusion of more than seven axes.

Kruskal–Wallis tests with eta^2^ statistics revealed a total of 16 statistically significant (*p* < 0.05) relationships between traits and functional axes (electronic supplementary material, tables S4, figure S3). The first functional axis (PC1) had the highest number of significant trait correlations (six), and this axis appears to be driven by all traits except mineralogy and habitat. Most of the significant trait-axis correlations occur on the first four functional axes (see electronic supplementary materials for a fuller discussion of the statistical procedures described above).

**Figure 3.**
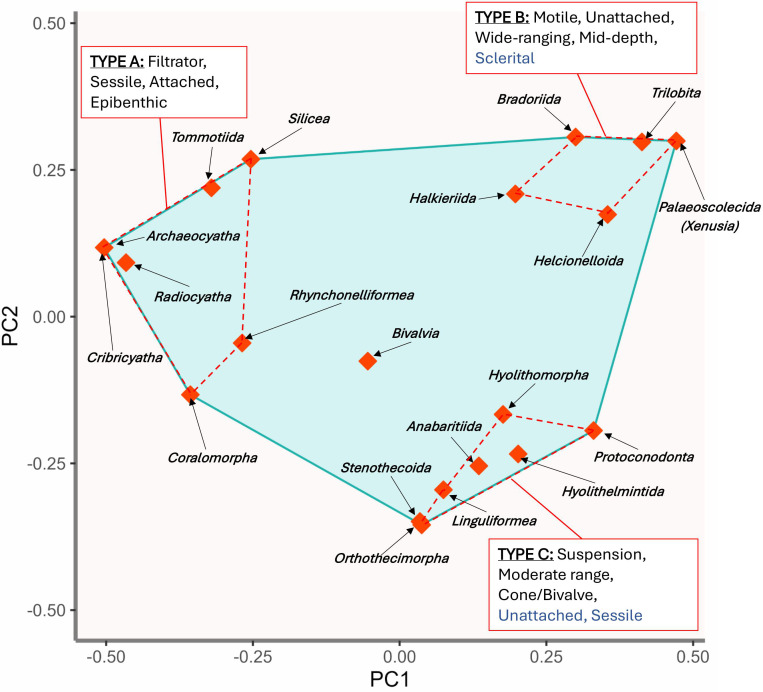
Convex hull of global functional space for Siberian Platform metazoan taxa from approximately 529 Ma to 508 Ma. Space is displayed against two functional axes: PC1 and PC2. Points represent positions of taxonomic groups based on their coordinates in seven-dimensional space. Taxonomic groups at the extremes of functional space are highlighted in three subjectively defined clusters (Types A, B and C) with shared trait modalities. Modalities in black text are common to all taxonomic groups in a cluster; modalities in blue text are common to all taxonomic groups in a cluster except one.

The convex hull of functional space for all taxonomic groups is displayed against two functional axes, PC1 and PC2 (figure 3), with points representing the positions of taxonomic groups based on their coordinates in the seven-dimensional space. Taxonomic groups at the extremes of functional space are highlighted in three subjectively defined clusters based on their proximity to one another in functional space (Types A, B and C). Type A (Archaeocyatha, Coralomorpha, Radiocyatha, Cribricyatha, Silicea, Tommotiida and rhynchonelliformean Brachiopoda) consists of sessile, epibenthic and attached, filter-feeders, several of which have heavily calcified (aragonite and high-Mg calcite) skeletons. Type B (Trilobita, Bradoriida, Palaeoscolecida, Halkieriida, Helcionelloida) are motile, unattached taxa tolerant of a wide range of depths and with diverse feeding strategies, with all groups (except Helcionelloida) having plated exoskeletons. Hyolithomorph hyoliths are assigned to the type B cluster of motile animals because they are associated with their own trace fossils and possess locomotory appendages (helens) although of an unusual structure [79,80]. Type C metazoans are mostly sessile suspension feeders with moderate depth ranges with have either conical, tooth-like or bivalved skeletons (Orthothecimorph Hyolitha, Anabaritida, Protoconodonta, Hyolithelminthida, linguliformean Brachiopoda, Bivalvia and Stenothecoida). All Type C taxa, except some linguliformeans, are also unattached.

### (c) Species richness, functional richness, functional dispersion and functional beta-diversity

**Figure 4.**
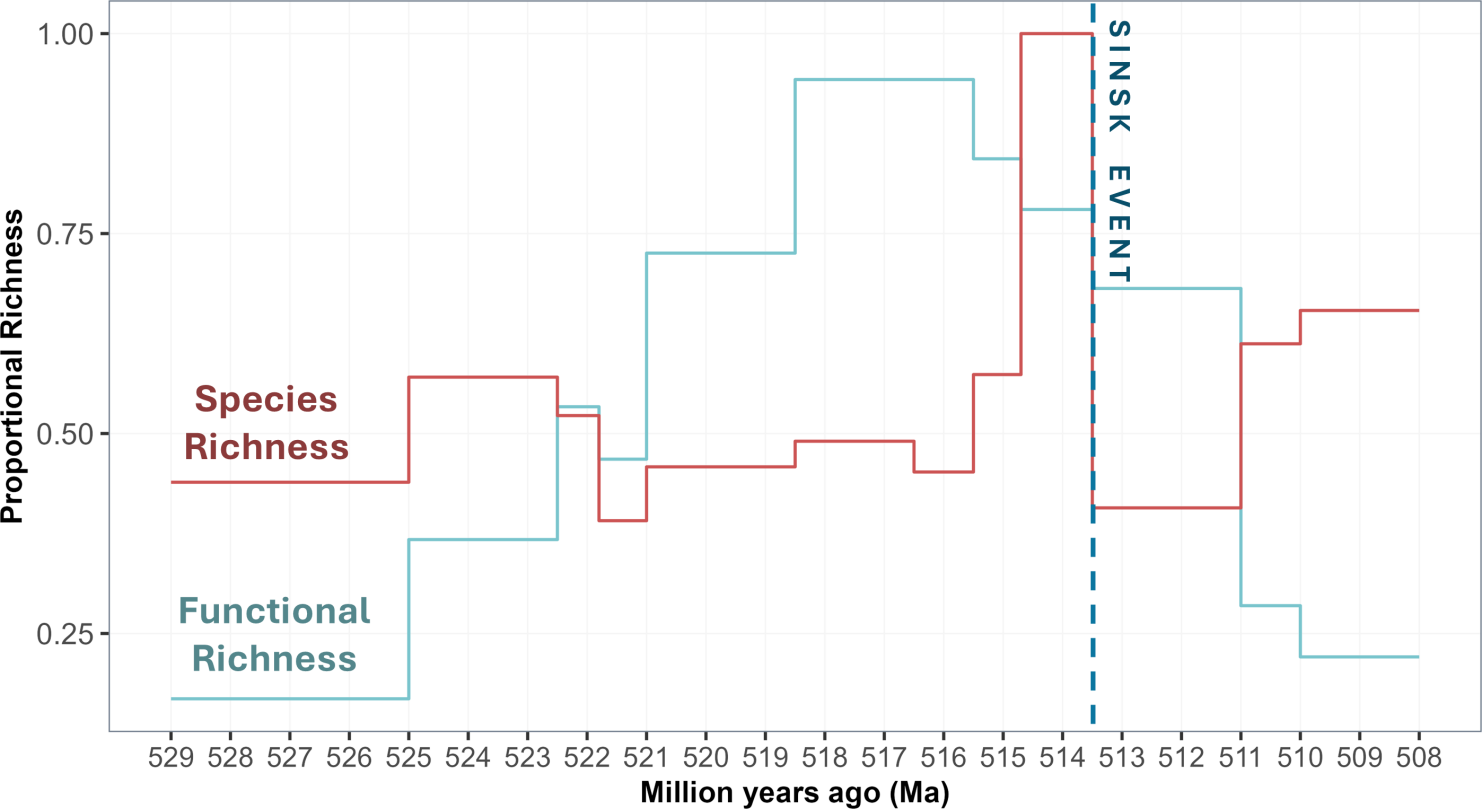
Species richness and functional richness for all metazoan taxonomic groups on the Siberian Platform, from approximately 529 Ma to 508 Ma, binned by biostratigraphic zones, with timing of Sinsk Event. Functional richness indices represent the proportional occupation of global functional space by each assemblage. Species richness is presented as proportionate to the maximum species richness in the study interval (*n* = 312 at approx. 514 Ma).

Species richness shows small scale variations from approximately 529 Ma to approximately 520 Ma, after which this dramatically increases until approximately 514 Ma (reaching *n* = 312 spp.), declines significantly at the Sinsk Event (*n* = 127 spp.), followed by a recovery thereafter (figure 4).

By contrast, functional richness begins low at approximately 529 Ma and increases rapidly until approximately 520 Ma. This increase is driven by the emergence of new taxonomic groups with novel trait combinations (e.g. trilobites, rhynchonelliformean brachiopods, palaeoscolecidans, bradoriids, stenothecoids and coralomorphs), despite overall species richness declining by 521 Ma remaining relatively stable thereafter (figure 4). After a peak at approximately 518–515.5 Ma, functional richness starts to decline while species richness increases rapidly. Functional richness continued to decline to even lower levels after the Sinsk Event, while species richness began to quickly recover in some groups.

**Figure 5.**
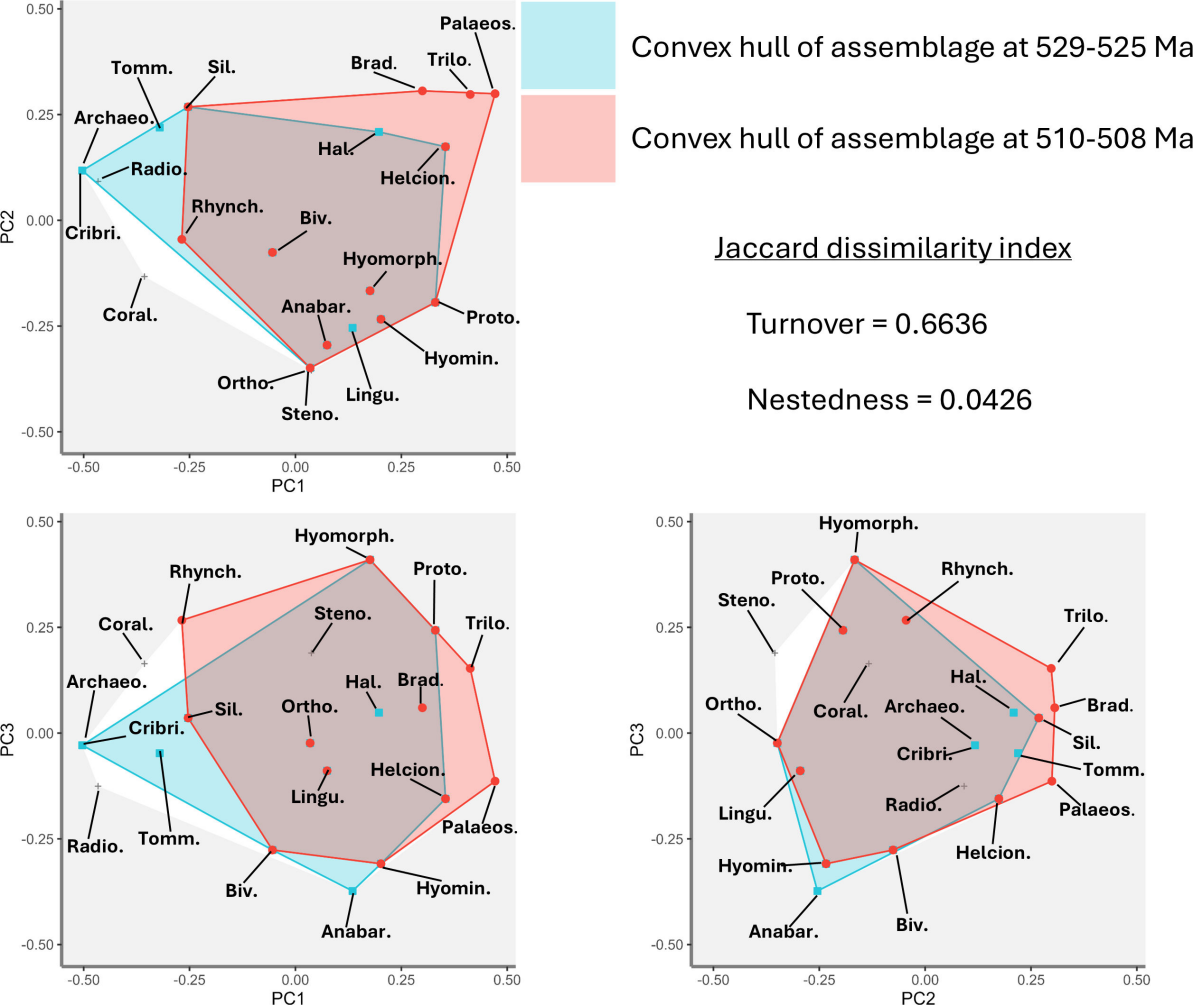
Functional beta diversity for axes PC1–PC3, comparing assemblages at the beginning (529–525 Ma) and end (510–508 Ma) of the study interval. Points represent the distribution of taxonomic groups in functional space. Convex hulls are stacked with the second time interval superimposed over the first. ‘Sil.’ = silicean sponges, ‘Tomm.’ = tommotiids, ‘Archaeo.’ = archaeocyaths, ‘Radio.’ = radiocyaths, ‘Cribri.’ = cribricyaths, ‘Coral.’ = coralomorphs, ‘Palaeos.’ = palaeoscolecidans & xenusians, ‘Brad.’ = bradoriids, ‘Trilo.’ = trilobites, ‘Hal.’ = halkieriids, ‘Helcion.’ = helcionelloids, ‘Biv.’ = bivalved molluscs, ‘Rhynch.’ = rhynchonelliformean brachiopods, ‘Lingu.’ = linguliformean brachiopods, ‘Hyomorph.’ = hyolithomorph hyoliths, ‘Ortho.’ = orthothecimorph hyoliths, ‘Anabar,’ = anabaritids, ‘Proto.’ = protoconodonts, ‘Hyomin.’ = hyolithelminthes, ‘Steno.’ = stenothecoids.

Functional beta-diversity was calculated for assemblages at 529 Ma and 508 Ma using the Jaccard dissimilarity index (as proposed by Villéger *et al*. [81]). The high turnover and low nestedness scores (figure 5) indicate that these two assemblages are highly functionally distinct, despite having similarly low functional richness values.

### (d) Changing occupation of functional space across the Sinsk Event

**Figure 6.**
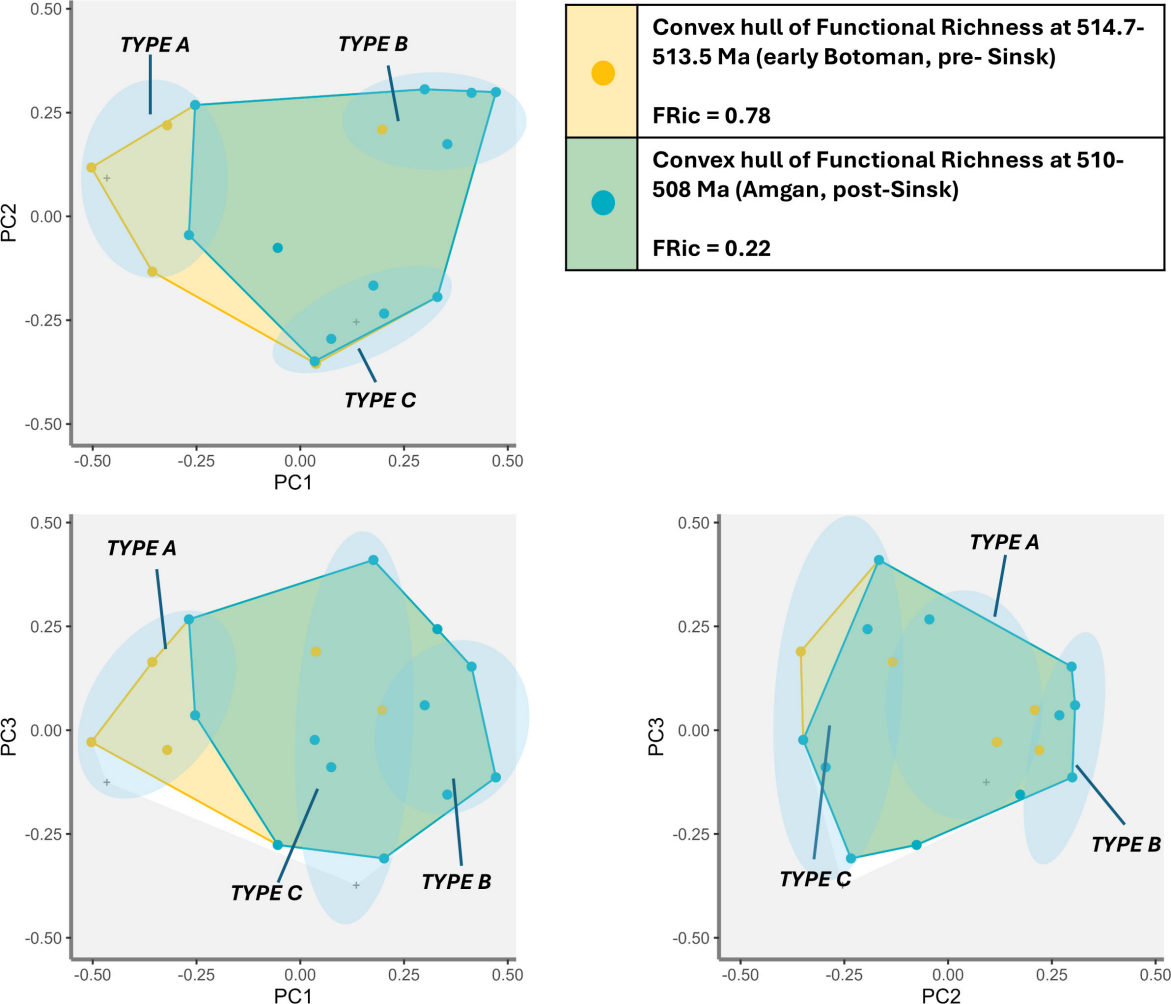
Functional richness for axes PC1–PC3 comparing time intervals pre-Sinsk (514.7−513.5 Ma) and post-Sinsk Event (510–508 Ma). Points represent the distribution of taxonomic groups in functional space. Approximate position of the three clusters (Types A, B and C) with common traits is shown. Convex hulls are stacked with the second time interval superimposed over the first.

Comparison of functional richness for the pre- and post-Sinsk intervals (figure 6) shows that taxonomic groups which were motile, tolerant of variable depth ranges and had diverse feeding strategies began to diversify in the aftermath of the Sinsk (mainly Type B cluster) while sessile, filter-feeding and heavily calcified (aragonite and high-Mg calcite) shallow marine specialists (mainly Type A cluster) which had been dominant prior to the extinction, failed to radiate.

**Figure 7.**
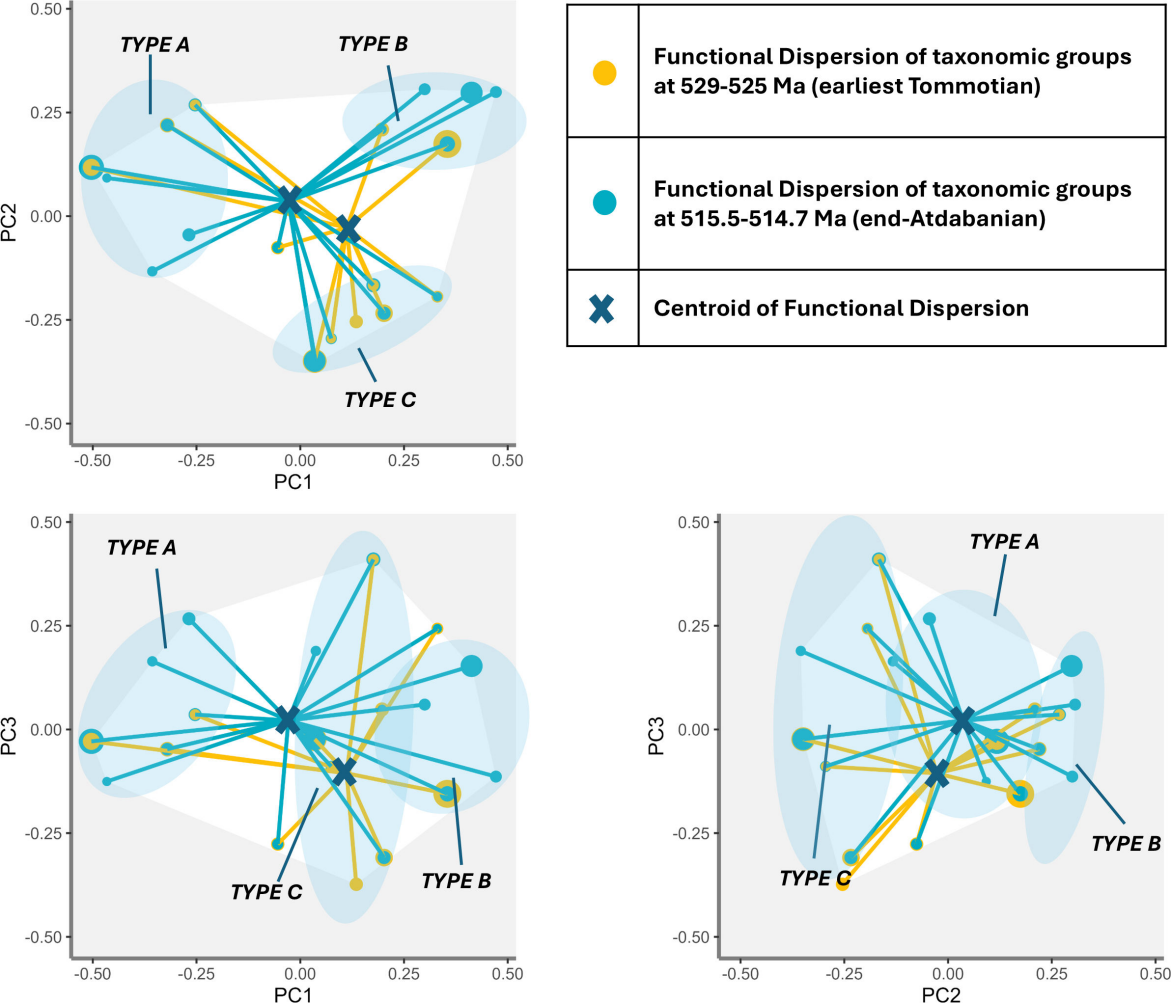
Functional dispersion for axes PC1–PC3 comparing assemblages at the beginning of the entire study interval (529–525 Ma) and pre-Sinsk Event (515.5−514.7 Ma). Points represent taxonomic groups in functional space, size of points represents relative weight (species richness) of each taxonomic group, intersection of lines represents centroid of mean trait values of assemblage weighted by species richness.

**Figure 8.**
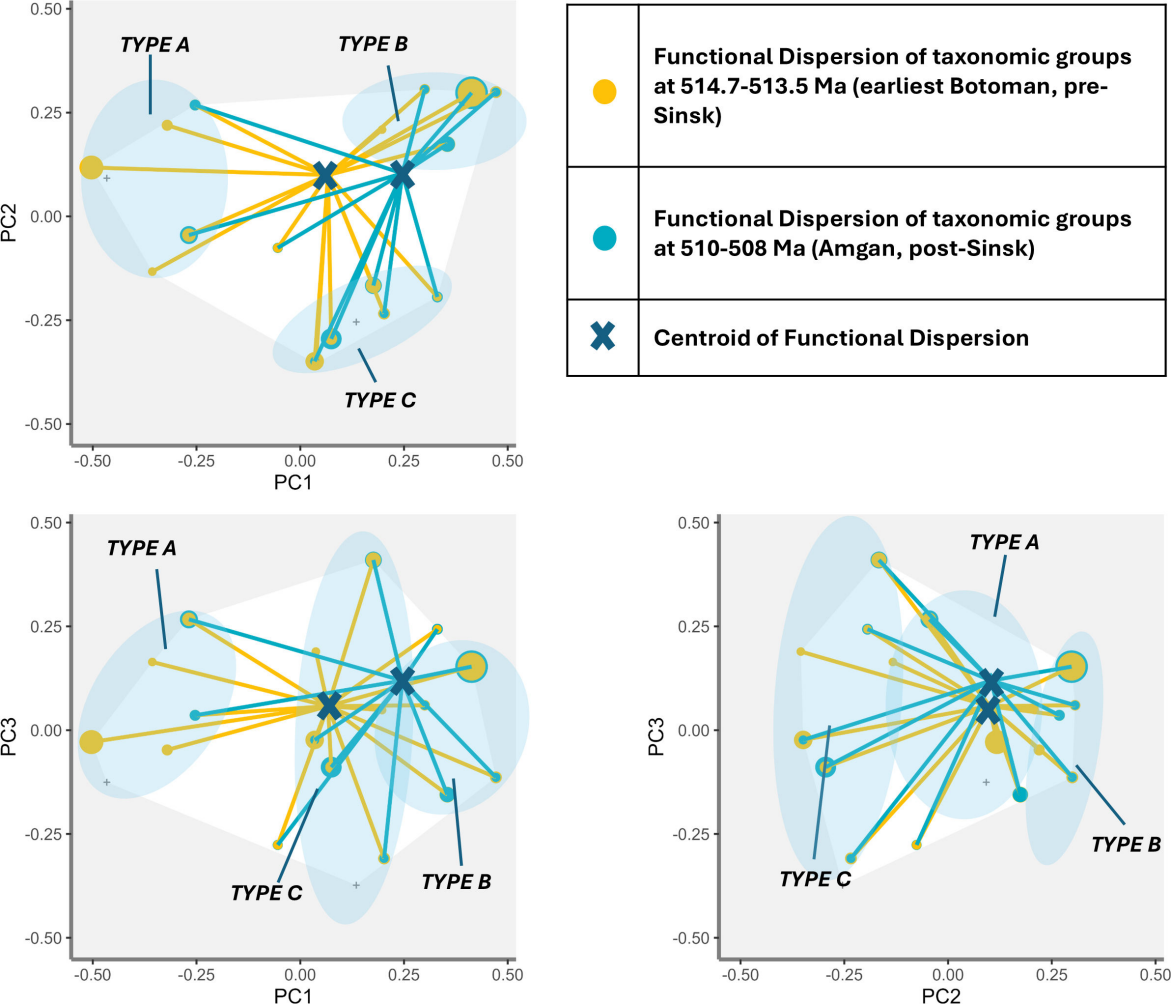
Functional dispersion for axes PC1–PC3 comparing assemblages immediately prior to (514.7−513.5 Ma) and in the aftermath of (510–508 Ma), the Sinsk Event. Points represent taxonomic groups in functional space, size of points represents relative weight (species richness) of each taxonomic group, intersection of lines represents centroid of mean trait values of assemblage weighted by species richness.

Functional dispersion pre-Sinsk (515.5−514.7 Ma) and post-Sinsk (510–508 Ma) shows that the centroid of functional dispersion pre-Sinsk is towards the Type A cluster coincident with the peak of functional richness. The centroid then began to shift towards the Type B cluster, as functional richness began to decline and species richness to rapidly increase. This shift continued unabated through the Sinsk Event until approximately 510 Ma, by which time the centroid has strongly shifted towards Type B taxonomic groups (figures 7 and 8).

## 4. Discussion

### (a) Sampling, preservation and taxonomic biases

In the lower Cambrian of the Siberian Platform, all metazoan fossils are restricted to fine-grained argillaceous limestones (mostly wackestones and packstones), and some grainstones, all of which accumulated onshore above either normal wave or storm wave base [7,15]. All the taxa considered here have been identified either from thin sections (archaeocyaths and other attached reef fauna), or on hand sample surfaces under a light microscope (e.g. trilobites, bradoriids, hyolithomorph hyoliths, brachiopods), or from the dissolution and disaggregation of whole rock samples (small shelly fossils). Small shelly fossils are represented by taxa of comparable millimetric sizes, forming part of the small shelly fauna as shells and disarticulated sclerites. These are generally preserved either as moulds or by secondary phosphate replacement. Only lingulate brachiopods and tommotiids are preserved as original shells, and of calcitic taxa only rhynchonelliform brachiopods retain their original low-Mg calcite mineralogy.

All small shelly fossils are extracted using the same method of dissolution in buffered acetic acid to isolate phosphatic and phosphatized shells, or moulds and steinkerns, so there is no bias towards particular taxa or groups. Researcher bias is also unlikely given that the assemblages reflect multiple and different studies over many decades, where no single worker or study dominates [15]. We infer therefore that preservational and taphonomic biases are minimized, and that any sampling biases present are shared equally by all small skeletal fossil groups.

Potential over-splitting at the species level might affect our estimates of overall species richness (figures 2A and 4), but we are confident that any errors are minimal. Functional traits are assigned at the level of broad taxonomic groups, and so our analyses of functional space occupation and functional richness are not affected.

### (b) Functional diversity and species richness before the Sinsk Event

We show that occupation of functional space expanded rapidly during the Cambrian Radiation from approximately 529 Ma and peaked during the approximately 518.5–515.5 Ma interval, thereafter entering a decline which was only mildly exacerbated by the Sinsk Event at approximately 513 Ma (figure 4). Species richness also increased with the occupation of functional space, though somewhat asynchronously: climbing rapidly during a brief interval from approximately 515.5–513.5 Ma before crashing at the Sinsk Event.

The increase in functional richness prior to the Sinsk Event is due to the emergence of new taxonomic groups with novel sets of functional traits, leading to the rapid expansion of functional space occupation from 529 Ma to 515 Ma, during which time species richness remained relatively stable. This may reflect the replacement of functionally similar taxonomic groups with more functionally differentiated ones during the Cambrian Radiation, as evidenced by originations and extinctions of taxonomic groups as functional diversity began to increase (figures 2B and 4). This could explain the apparent lag between increases in functional and species richness, since new body plans and functional trait combinations had first to emerge, before subsequent diversification within those taxonomic groups. An oxic pulse is inferred to have occurred on the Siberian Platform at approximately 515 Ma [4], and this may have presented a period of favourable or expanded habitable conditions which permitted rapid diversification in these newly emerged taxonomic groups.

During the Radiation, from approximately 529 Ma to approximately 520 Ma, most taxonomic groups share many traits in common with archaeocyaths—they are sessile, epibenthic, filter—or suspension-feeding metazoans that preferentially occupied shallow waters. The centroid of functional dispersion moves significantly closer to Type B taxonomic groups from approximately 515.5 Ma to 508 Ma (figures 7 and 8), representing a move towards those groups which are motile, unattached, have wider depth ranges, often occur in deeper waters and have diversified feeding strategies. This change is largely driven by trilobite diversification—though there are also smaller increases in the species richness of Type B helcionelloids and palaeoscolecidans during this period.

A transition from metazoan communities dominated by a sessile benthos to more ecologically complex ecosystems with diverse guilds of motile predators and scavengers is also characteristic of the Great Ordovician Biodiversification Event (GOBE) [82], and a similar transition may already have occurred in the early Cambrian Siberian Platform. Species diversity during the Phanerozoic has been noted to generally increase with the occupation of new areas of ecospace, which is broadly synonymous with functional space [34]. Siberian Platform palaeocommunities exhibited a similar pattern prior to the Sinsk Event, but that this process was asynchronous and the increase in functional richness preceded the increase in species richness—a pattern perhaps only detectable owing to the relatively high-resolution temporal record examined herein.

### (c) Non-selectivity at the onset of the Sinsk Event, and decoupling of taxonomic and functional diversity

The Sinsk Event resulted in a fundamental re-organization of the taxonomic composition of shallow marine metazoan communities. Archaeocyaths, halkieriids, stenothecoids and coralomorphs were decimated to become extinct shortly thereafter, but trilobites, helcionelloids, brachiopods, protoconodonts and hyoliths diversified, even though all of these groups underwent a substantial reduction in diversity at the Sinsk. Silicean sponges diversified during the immediate aftermath of the Sinsk [15]. Other remaining groups survived, but at very low diversities (1–3 species per group).

While relatively species-rich groups (including archaeocyaths, trilobites, hyoliths and helcionelloids) were decimated by the Sinsk Event, these groups have little in common with respect to their distribution in functional space. This indicates that the initial impacts of the extinction were non-selective with respect to functional traits. It is possible that the Sinsk Event represented such a severe environmental perturbation (i.e. deoxygenation of the shallow shelf habitat) that no preferential survival benefits were conferred by particular functional traits. All these groups, however, were dominated by endemic species and genera with short longevities, which are known to be more prone to extinctions [11,14,83].

Species richness entered another phase of rapid recovery approximately 2.5–5 Myr after the Sinsk Event, during which time functional richness continued to decline towards its nadir. This suggests that taxonomic and functional diversity may have become decoupled in the wake of the Sinsk Event. By 508 Ma functional and species richness had returned to levels similar to those found at approximately 529 Ma, though the region of functional space occupied had shifted dramatically indicating that new taxonomic groups with novel functional traits had become dominant. This suggests a delayed ecological impact of the Sinsk Event: richness within taxonomic groups was reduced as species went extinct during the Sinsk, and taxonomic groups with reduced species richness were more vulnerable to disappearing due to subsequent species extinctions. These extinctions occurred while other taxonomic groups were diversifying, creating an inverse relationship between functional and species richness.

This pattern is consistent with other studies which have reported decoupling of functional and taxonomic diversity in the wake of mass extinction events, e.g. the Permian–Triassic mass extinction [38,41,42,84], and among corals during the Late Devonian [43]. The end-Ordovician (Hirnantian) mass extinction, while more protracted than the Sinsk Event, also decimated reef communities and mostly affected tropical faunas, which were replaced by similar, but less diverse, cool-water communities [85–87]. Both the Sinsk and end-Ordovician events saw a notable diversification of siliceous sponges, which appear to have been able to flourish under the low oxygen conditions that developed during global warming [85,88].

### (d) Selectivity in the aftermath of the Sinsk Event: particular trait combinations facilitate post-extinction recovery

While the immediate impacts of the Sinsk Event appear to have been non-selective with respect to functional traits, by contrast the recovery and extinction in the aftermath of the Sinsk appears to have been highly selective. We find that those taxonomic groups with heavily calcified and massive skeletons that were reef-associated had completely disappeared by approximately 510–508 Ma; most notably the previously highly diverse archaeocyaths.

Skeletal mineralogy and type traits have been proposed to be critical determinates of extinction during some mass extinctions driven, at least in part, by severe anoxia and hypercapnia—85% of organisms with massive calcium carbonate skeletons were lost in the Permian–Triassic mass extinction [74,89]. This is in contrast to the fate of silicean sponges, which diversified during the immediate aftermath of the Sinsk Event [15]. This pattern is consistent with the observation that some modern siliceous demosponges are able to tolerate remarkably low concentrations of dissolved oxygen [90].

The taxonomic groups which begin to recover after the Sinsk Event were those that were generally unattached and motile, occupied a wide or moderate range of depths rather than shallow-water specialists, and with sclerital exoskeletons or bivalved shells. Most groups that occupied shallow-water depths with narrow-ranges, were attached, reef-building or with conical shells, did not recover. Indeed, rhynchonelliformean and linguliformean brachiopods are the only groups of sessile benthos to recover significantly. There is also a marked increase in nektonic and predatory or scavenging species (mostly trilobites, although there is also a minor increase in protoconodonts and palaeoscolecidan worms).

Many of the groups which began to recover species richness post-Sinsk exhibited within-group diversity in individual traits (referred to herein as ‘composite modalities’) for feeding mode (trilobites, helcionelloids, hyolithomorph hyoliths), substrate relationships (linguliformean brachiopods), as well as habitat (trilobites, helcionelloids and linguliformean brachiopods). Of the four groups which became extinct after the Sinsk Event, none possessed composite modalities for any trait. This suggests that taxonomic groups possessing within-group diverse feeding strategies and habitat associations—together with an unattached and motile habit, and a wide tolerance of water depth or occurrence at mid-depths—were able to diversify after the Sinsk Event, while those groups which did not possess these traits either remained at either very low species diversities or became extinct. Some of these ‘composite modalities’ can be considered as generalist traits.

The aftermath of the Sinsk Event therefore constitutes the beginning of an interval of ecosystem restructuring, as both extinction and diversification appears highly selective and is well-explained by functional trait combinations. It seems likely that the initial onset of the Sinsk Event constituted an environmental perturbation of such severity that all taxonomic groups underwent a significant loss in species diversity, irrespective of their individual trait combinations, most likely with anoxia combined with elevated temperatures as the primary kill mechanisms. It is unknown whether adverse environmental conditions continued in the aftermath of the Sinsk (approx. 511 Ma to 508 Ma), but taxonomic groups with a particular suite of functional traits were able to persist and begin rapidly diversifying, while others eventually succumbed entirely.

In sum, we show that certain functional traits appear to have been instrumental in extinction selectivity and survival on the Siberian Platform. This confirms that the Sinsk Event marks a significant transition in the Cambrian Radiation: a point after which Siberian Platform communities returned to the low functional richness characteristic of the earliest Cambrian, but dominated by taxonomic groups with distinctly novel functional attributes.

## Data Availability

All data used in analyses and for the generation of figures is publicly available in the Dryad repository, along with fully annotated R scripts to facilitate reproduction [91]. Supplementary material is available online [92].
